# The Practitioner’s Ready Reference Book, a Handy Guide in Office and Bed-side Practice

**Published:** 1883-06

**Authors:** 


**The Practitioner's Ready Reference Book, a handy guide in office and bed-side practice.** By RICHARD J. DUNGLISON, A. M., M. D., Editor of "Dunglison's Medical Dictionary," etc. Third edition, thoroughly revised and enlarged. Philadelphia: P. Blakiston, Son & Co. 1883.

It is less than three years ago that the second edition of this book was received. The issue of a third edition in so short a time proves that the work is fully appreciated by the profession. To those who have not read it we will say that it is a work of ready reference, containing, in a compact and tangible shape, a vast amount of practical information. This new edition is in many ways a great improvement upon the last, and those who are wise enough to add it to their libraries, will find the consultation of its pages almost an every day occurrence.

